# Expectations, needs and mid-term outcomes in people accessing to secondary findings from ES: 1st French mixed study (FIND Study)

**DOI:** 10.1038/s41431-024-01616-9

**Published:** 2024-05-27

**Authors:** Eléonore Viora-Dupont, Françoise Robert, Aline Chassagne, Aurore Pélissier, Stéphanie Staraci, Damien Sanlaville, Patrick Edery, Gaetan Lesca, Audrey Putoux, Linda Pons, Amandine Cadenes, Amandine Baurand, Caroline Sawka, Geoffrey Bertolone, Myrtille Spetchian, Meriem Yousfi, Dominique Salvi, Elodie Gautier, Antonio Vitobello, Anne-Sophie Denommé-Pichon, Ange-Line Bruel, Frédéric Tran Mau-Them, Anne Faudet, Boris Keren, Audrey Labalme, Nicolas Chatron, Carine Abel, Sophie Dupuis-Girod, Alice Poisson, Julien Buratti, Cyril Mignot, Alexandra Afenjar, Sandra Whalen, Perrine Charles, Solveig Heide, Linda Mouthon, Sébastien Moutton, Arthur Sorlin, Sophie Nambot, Anne-Sophie Briffaut, Marie-Laure Asensio, Christophe Philippe, Christel Thauvin-Robinet, Delphine Héron, Massimiliano Rossi, Nicolas Meunier-Bellard, Marcela Gargiulo, Christine Peyron, Christine Binquet, Laurence Faivre

**Affiliations:** 1 FHU TRANSLAD, GAD INSERM UMR 1231, University of Burgundy, Dijon, France; 2 Genetics Department, Reference Center for Developmental Disorders, University Hospital, Dijon, France; 3 Genetics Department, Reference Center for Developmental Disorders, HCL, Bron, France; 4 Clinical Psychology Lab., Psychopathology, Psychoanalysis (EA4056, ED 261), University of Paris, Sorbonne Paris City, Paris, France; 5 Laboratory of Sociology and Anthropology (LaSA, EA3189), University of Burgundy-Franche-Comté, Besançon, France; 6 Laboratory of economy (LEDi), University of Burgundy, Dijon, France; 7 Genetics Department, Reference Center for Hereditary Cardiac Disorders, GH APHP, Paris, France; 8 Univ Lyon, Univ Lyon 1, CNRS, INSERM, Physiopathologie et Génétique du Neurone et du Muscle, UMR5261, U1315, Institut NeuroMyoGène, 69008 Lyon, France; 9 INSERM U1028, CNRS UMR5292, CRNL, GENDEV Team, University of Claude Bernard Lyon 1, Bron, France; 10 Genetics Department, Reference Center for Developmental Disorders, GH APHP, Paris, France; 11 Reference Center for Rare Disorders with psychiatric expression C.H. Le Vinatier, Bron, France; 12 Equipe de recherche AESIO santé, unité de Sant Etienne, Clinique médico chirurgicale mutualiste, Saint Etienne, France; 13 CHU Dijon Bourgogne, INSERM, Université de Bourgogne, CIC 1432, Module Épidémiologie Clinique, Dijon, France; 14 Genetics Department, Reference Center for Intellectual Disabilities, University Hospital, Dijon, France; 15 Institute of myology, GH APHP, Paris, France

**Keywords:** Genetics research, Genetic counselling, Psychology, Ethics, Medical ethics

## Abstract

Generation and subsequently accessibility of secondary findings (SF) in diagnostic practice is a subject of debate around the world and particularly in Europe. The French FIND study has been set up to assess patient/parent expectations regarding SF from exome sequencing (ES) and to collect their real-life experience until 1 year after the delivery of results. 340 patients who had ES for undiagnosed developmental disorders were included in this multicenter mixed study (quantitative *N* = 340; qualitative *N* = 26). Three groups of actionable SF were rendered: predisposition to late-onset actionable diseases; genetic counseling; pharmacogenomics. Participants expressed strong interest in obtaining SF and a high satisfaction level when a SF is reported. The medical actionability of the SF reinforced parents’ sense of taking action for their child and was seen as an opportunity. While we observed no serious psychological concerns, we showed that these results could have psychological consequences, in particular for late-onset actionable diseases SF, within families already dealing with rare diseases. This study shows that participants remain in favor of accessing SF despite the potential psychological, care, and lifestyle impacts, which are difficult to anticipate. The establishment of a management protocol, including the support of a multidisciplinary team, would be necessary if national policy allows the reporting of these data.

## Introduction

Next generation sequencing offers new diagnostic opportunities in the context of rare diseases [1]. It leads to a primary finding (PF) more frequently than other conventional strategies, but it can also reveal variants unrelated to the patient’s disease. These findings are either “incidental” when they are discovered by chance (IF), or “secondary” if they are actively sought (SF). Their discovery can lead to preventive or therapeutic action as part of a personalized medicine approach. The aim is to prevent the onset of disease, and to reduce morbidity and mortality. In 2013, the American College of Medical Genetics and Genomics (ACMG) defined a list of 56 genes accessible for prevention and/or treatment, including genes predisposing to cancer, cardiovascular and metabolic diseases. This list is regularly revised, most recently to 81 genes in 2023 [2–6]. Generation and subsequently accessibility of secondary findings (SF) remains a matter of debate in France and elsewhere. The European Society of Human Genetic and Canadian College of Medical Genetic have recommended that IF/SF not be made available until evidence of medical utility is demonstrated [7–10].

The international literature, in particular from the US, includes several quantitative and qualitative studies of patients, mostly in hypothetical situations, but less often in a context where the IF/SF are actually reported to patients [11–14]. All of these studies indicate that there is a broad desire to be informed of such results, whatever their category, to make life choices guided by this medical information. Conversely, professionals stress the risk of psychological consequences and the importance of establishing national recommendations, particularly for the definition of the “actionability” of genes [15–17].

Here we present our mixed study reporting the expectations, needs, and mid-term outcomes in patients/parents who chose to access SF after undergoing ES.

## Materials and methods

### General study design

The FIND study, conceived by a multidisciplinary team (geneticists, genetic counselors, psychologists, health economists, ethicists, sociologists, anthropologists, epidemiologists, patients’ associations), uses a mixed design associating qualitative and quantitative methods. The FIND study received approval from the local ethics committee (N°: 17-DIJO-01, 11/10/2017), and all participants provided consent for the use of research data in an anonymous manner.

### List of studied secondary findings

In this project, SF was defined, according to ACMG and the French *Agence de la Biomédecine* (ABM) [18] recommendations, as pathogenic or probably pathogenic genomic variants (classes 5 and 4), that they have no direct relationship with the initial indication or, for phamacogenetic, variant-drug combinations with strong scientific evidence, that are actively sought by analyzing a pre-established list of genes. The methodology for interpreting SF has already been published [19]. Three actionable groups were considered (Supplementary Table 1): (i) *1st* *SF category 1 (SF1):* variants involved in a genetic predisposition to a preventable or treatable disease, mostly occurring in adulthood, using the list of 118 genes proposed by Dorschner [20], including the ACMG list of 59 genes [2, 3], plus four genes involved in bleeding disorders; (ii) *2nd* *SF category (SF2):* variants in 114 genes with an impact on genetic counseling for future pregnancies, including heterozygous carriers of a recessive disease whose prevalence of heterozygotes would lead to screening of the partner in a given population and female carriers of an X-linked recessive disease; (iii) *3rd* *SF category (SF3):* two genes whose homozygous or compound heterozygous pharmacogenomic variants have a clinical interest for drug adaptation in the context of neurodevelopmental disorders.

**Fig. 1:**
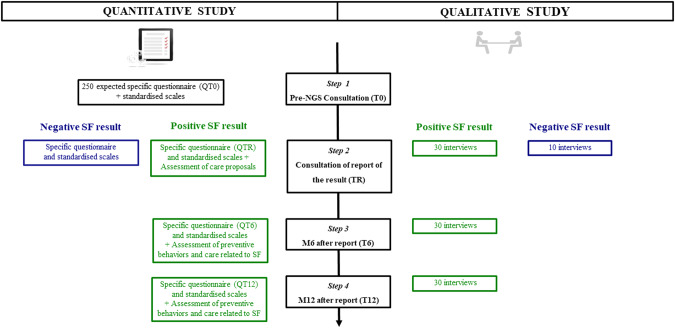
Expected study design of the FIND study. A mixed methodology was proposed, including both a quantitative and qualitative study. For the quantitative study, all participants had to fill in specific and standardized questionnaires at inclusion (QT0). In order to have 250 respondents, with a 25–30% risk of being lost to follow-up, the number of subjects to include was 330. They also had to fill in questionnaires at time of results (QTR), 6 months after results (QT6), and 12 months after results (QT12). QT0: study data concerning the perception of the interest of SF, the needs and expectations regarding the information given about SF, the selected SF category, as well as the reasons for these choices. QTR - QT6 – QT12: The self-administered questionnaire consisted of closed multiple-choice questions and visual analog scales (VAS) that ranged from 0 (lowest agreement or satisfaction score) to 100 (highest agreement or satisfaction score). Standardized scale: anxiety (STAI-Y A and B), depression (CES-D), and quality of life (QoL; SF12 mental and physical). For the qualitative study, the same 30 participants with SF+ were asked to participate at TR, T6, T12, and 10 participants with negative results were planned to be interviewed at TR. Participants also filled in the questionnaires.

### Study course (Fig. 1)

Fetuses, children, and adults with developmental abnormalities requiring solo ES in order to identify the causal diagnosis were recruited from October 2017 to March 2019 in three French genetic clinics (Dijon, Lyon, and Paris). Participants surveyed were the parents and all included adult index cases able to understand, speak French and consent to the study. The geneticist informed the patient/the parents about the project and about the search for SF. If they were interested in participating in the study or having additional information regarding SF to make their decision, they systematically met with a genetic counselor specifically trained in genomics. At the end of this information process, within a 1-month period, participants confirmed their desire to access SF or not (SF1, 2 and/or 3) and therefore their inclusion in the study. If they did not wish to access SF results, participants were asked what led them to their decision. Participants could change their mind at any time until the results were reported. The inclusion could not be confirmed if one parent did not wish to access SF.

*First assessment (inclusion):* in the month following inclusion, all included participants were asked to complete standardized scales assessing anxiety (STAI-Y A and B) [21], depression (CES-D) [22] and quality of life (QoL ; SF12 mental and physical) [23], administered at each assessment, and self- administered questionnaires (QT0) (paper or online) to collect socio-demographic data, clinical data, and expectations regarding SF (based on multiple-answer questions).

*Second assessment (ES and SF results delivery—time of report (TR))* When ES and SF results were made available, a consultation with the geneticist was scheduled for each patient. During the consultation, the geneticist first reported the PF and secondly, if participants confirmed their choice to access SF, the SF results were delivered by the geneticist and/or the genetic counselor, assisted or not by another specialized physician. Then participants completed the same three standardized scales of anxiety, depression and quality of life and a specific self-administered questionnaire (QTR) according to the nature of their result (with or without PF, with or without SF, and according to the SF category). These questionnaires, based on multiple-answer questions, explored the reasons behind their choice to access SF, possible changes of opinion, their experience and satisfaction with the results obtained and the information and support received.

Thirty parents of minors or of protected adults with a SF (SF+) (all SF group 1 and a selection of SF groups 2/3) and ten without SF (SF-) were asked to participate in a semi-directive interview with one of two training psychologists or one training sociologist according to center in order to evaluate their feelings after the results were reported.

It was decided that this research would focus primarily on the experiences of parents/patients who had received a SF1 (predisposition to late-onset actionable diseases), by offering an interview to all patients, given their limited number, and their major interest since they include the minimum list defined by the ACMG. Thus a selection of files were made for patients with SF2 (genetic counseling) or SF3 (pharmacogenomics), taking care to explore a variety of situations: Patients with a positive or negative primary diagnosis; SF diversity (e.g., CFTR and HFE for SF2); minor and adult patients; mother or father interviewed.

The interview guide was developed jointly by a sociologist and a psychologist following their SEQUAPRE experience [11]. Most of the first interviews took place in person following the consultation dedicated to the delivery of results of ES.

*Follow-up assessments (6 and 12 months after delivery of results—T6 and T12)* Each individual with SF+ was asked to complete specific self-administered questionnaires in addition to the same standardized scales of anxiety, depression and quality of life, at 6 and 12 months after the delivery of results (QT6 and QT12). These questionnaires explored the experience at a distance from the results, the perception of the interest of the results, the satisfaction, the needs and expectations in terms of support, the level of appropriation of the results, and the consequences of the results for the patient and his family in terms of prevention and care, basing on multiple-answer questions. The same parent of individuals with SF+ was interviewed at each timepoint in order to explore how the impact of SF results on the individual and the family changed over time.

### Data collection and treatment

All the questionnaires were anonymized and entered in a specific database via a Clinical Data Management System (Cleanweb®, Telemedecine Technologies, Boulogne-Billancourt, France) managed by data managers from CIC-EC1432. The interviews were recorded, transcribed in full and anonymized, and then analyzed after several in-depth readings to identify emerging themes that were encoded and analyzed with the NVivo software (version 11). To reduce analysis bias, the first five interviews were analyzed and coded jointly by a sociologist and a psychologist trained in qualitative analysis.

A first conceptual thematic encoding was carried out based on the four concepts emerging from the literature review: psychological effects of the announcement, projection into the future, effect on parenthood, repercussions on lifestyle. Sub-themes then rapidly emerged and a final level of sub-themes was sometimes necessary.

### Statistical analysis

Continuous variables were expressed as means ± standard deviations (SD) or medians and minimums-maximums according to their distribution, and categorical variables as frequencies and percentages. At each time, means were compared using Kruskal-Wallis analysis of variance test when appropriate. Normality of variable distribution was checked with Shapiro-Wilks tests. Proportions were compared using Chi-squared or Fisher’s exact test when required. Longitudinal analyses of depression, anxiety, and quality of life scores were performed using linear nested random-effect models.

A *p* < 0.05 was considered statistically significant. Analyses were performed using SAS ™v9.4 (SAS Institute, USA) and Stata v15.1 (StataCorp LLC, USA).

## Results

### Characteristics of the population included

**Fig. 2:**
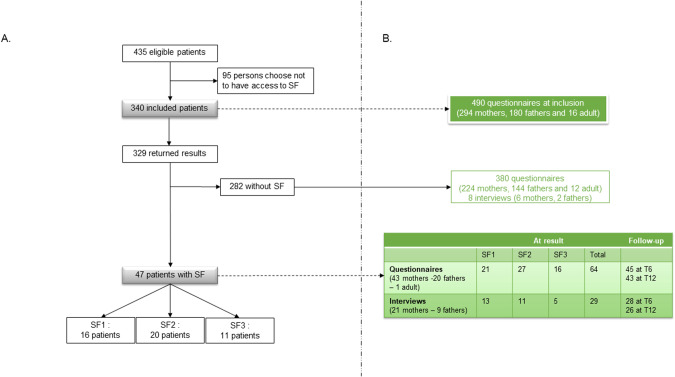
Flow chart of patients and participants. **A** This figure shows the number of patients eligible for the study and the number of people who wished to access their SF results. For people with SF, the SF category is specified; **B** Number of completed questionnaires and interviews *(results of questionnaires and interviews with parents without SF are presented in* Supplementary Table 3).

Over the study period, 435 patients undergoing ES were given the option of adding a search for SF. Finally, 78% (340 patients) requested to access SF (Fig. 2 and Supplementary Table 2).

Included patients were an average age of 13 ± 11 years and 56% were males. They presented with intellectual developmental disorder (IDD) (71%) and/or malformations (72%), and the majority (73%) had undergone previous genetic investigations. The characteristics of participants who completed questionnaires are presented in Table 1. Sixteen adult patients including six with learning disabilities (all interrogated independently from their parents), 294 mothers and 180 fathers completed at least one questionnaire. The level of education was at least equal to a high school diploma in 61% of mothers and 59% of fathers. One third of the mothers were not working, 76% by choice. Among the parents who were a couple (72%), 25% were in a precarious socio-economic situation (Epices score above 30). Among single parents, 58% of mothers and 29% of fathers were in a precarious situation. The level of situational anxiety was higher among mothers (27%) than among fathers (15%) (*p* = 0.003).

**Table 1:** Characteristics of participants and results of standardized and specific questionnaires at inclusion (*n* = 490).

			Mother	Father	Adult patient
**SOCIAL and DEMOGRAPHIC INFORMATION**			*n* = 294	*n* = 180	*n* = 16
Age (Median)			42 ± 10 years	43 ± 11 years	28 ± 13
Diploma	No diploma		18/276 (7%)	8/165 (5%)	4/14 (29%)
	Below the high school diploma		88/276 (32%)	60/165 (36%)	8/14 (57%)
	High school diploma		50/276 (18%)	27/165 (16%)	1/14 (7%)
	Bachelor’s degree		120/276 (43%)	70/165 (43%)	1/14 (7%)
Job	Working	Farmers	3/187 (1.6%)	6/143 (4.2%)	0/6
		Laborers	9/187 (4.8%)	35/143 (24.5%)	0/8
		Employees	104/187 (55.6%)	39/143 (27.3%)	4/6 (66%)
		Intermediate professions	20/187 (10.7%)	13/143 (9.1%)	1/6 (17%)
		Independent professions	7/187 (3.7%)	18/143 (12.6%)	0/6
		Managers and higher intellectual professions	44/187 (23.5%)	32/143 (22.4%)	1/6 (17%)
		*Total*	*187/294 (66%)*	*143/168 (85%)*	*6/14 (43%)*
	Not working	Personal choice not to work	66/87 (76%)	8/25 (32%)	7/8 (88%)
		Unemployed	9/87 (10.3%)	8/25 (32%)	0
		Retired	12/87 (13.8%)	9/25 (36%)	0
		*Total*	*95/294 (34%) (ND* = *8)*	*25/168 (15%)*	*8/14 (57%)*
**STANDARDIZED SCALES**					
Epices score (number and % of respondents with a score above 30)^a^			Single: 29/50 (58.0%)	Single: 5/17 (29.4%)	6/16 (37.5%)
			In couple: 62/245 (25.3%)		
SF12 (0–100) (Median (Minimum-Maximum))^b^		Physical scale	53 (23–66)	55 (18–67)	46 (31–61)
		Mental scale	43 (24–71)	47 (25–73)	45 (30–56)
CES-D (score above 23 for mothers and 17 for fathers)^c^			42/258 (16.3%)	18/158 (11.4%)	5/17 (29.4%)
STAI-Y (score above 46)^d^		Condition scale	69/253 (27.3%)	23/155 (14.8%)	2/17 (11.8%)
		Trait scale	79/253 (31.2%)	26/155 (16.7%)	8/16 (50.0%)
**SPECIFIC QUESTIONNAIRES**					
Assistance in making decisions about accessing to SF (multiple choice)		Geneticist	171/294 (58%)	99/180 (55%)	5/18 (28%)
		Alone	96/294 (33%)	63/180 (35%)	12/18 (67%)
		Partner or family	47/294 (16%)	35/180 (19%)	3/18 (17%)
Expectations (multiple choice)		Access to preventive measures	199/250 (79.6%)	120/160 (75.0%)	9/14 (64.3%)
		Prevent possible risks	181/250 (72.4%)	119/160 (74.4%)	11/14 (78.6%)
		Be better anticipate the future	198/250 (79.2%)	117/160 (73.1%)	11/14 (78.6%)
		Improve personal health status or that of their child	189/250 (75.6%)	127/160 (79.4%)	9/14 (64.3%)
		Have access to treatment	125/250 (50.0%)	74/160 (46.3%)	8/14 (57.1%)
		Adapt a treatment	115/250 (46.0%)	80/160 (50.0%)	4/14 (28.6%)
Report of SF for conditions with no treatment or preventive options (hypothetic situation)		In favor	105/294 (35.7%)	77/180 (42.8%)	6/18 (33.3%)
		Not in favor	108/294 (36.7%)	53/180 (29.4%)	11/18 (61.1%)
		No opinion	81/294 (27.6%)	50/180 (27.8%)	1/18 (5.6%)
Report of SF with uncertain status (hypothetic situation)		In favor	110/294 (37.8%)	81/179 (45.3%)	5/18 (27.8%)
		Not in favor	93/294 (32.0%)	48/179 (26.8%)	12/18 (66.7%)
		No opinion	88/294 (30.2%)	50/179 (27.9%)	1/18 (5.6%)

^a^The EPICES Score: The EPICES score (Evaluation de la Précarité et des Inégalités de santé dans les Centres d’Examens de Santé) is an individual indicator of precariousness for a single parent or an indicator per household. This self-administered questionnaire of 42 questions takes into account several dimensions of precariousness: employment, income, level of education, socio-professional categories, housing, family composition, social ties, financial difficulties, life events, perceived health. Statistical methods of factorial correspondence analysis and multiple regression were used to select 11 questions from the 42 questions that summarize 90% of a subject’s precariousness. The answer to each question is assigned a coefficient, and the sum of the 11 answers gives the EPICES score. The score is continuous, varyinf from 0 (not precarious) to 100 (maximum precariousness). The threshold of 30 is considered as the threshold of precariousness according to EPICES, the higher it is, the greater the precariousness. The reference study by Sass et al. [41] was used.

^b^The Short Form 12 or SF-12: The SF-12 is a self-assessment scale for quality of life, a complex concept to evaluate since it is characterized by its multi-dimensionality (physical, functional, emotional, spiritual and social well-being) and by subjectivity (it can only be correctly understood from the patient’s perspective). Designed for clinical research, the SF-12 is recognized as a practical and reliable alternative to the SF-36 in the nine European countries where it has been analyzed (Denmark, France, Germany, Italy, Netherlands, Norway, Spain, Sweden and the United Kingdom). It provides two scores: a mental quality of life score and a physical quality of life score and includes 12 items, divided into the same 8 dimensions as the SF-36: Physical activity; Life and relationships with others; Physical pain; Perceived health; Vitality; Limitations due to mental state; Limitations due to physical state; Psychological health. The maximum score is 100, the higher the score, the better the quality of life. In reference to Gandek et al. [23], the average reference score for the French population are 59.2 for the average SF-12 Physical score and 48.4 for the average SF-12 Mental score. In the 45-64 age group, the mean SF-12 Physical score was 49.4 and the mean SF-12 Mental score was 48.6. When the calculated scores of the parents are lower than these average reference scores, it means that the parents surveyed feel poorer physical and/or mental health than the average French person, and the opposite is true when their calculated scores are higher.

^c^CES-D: The Center for Epidemiologic Studies-Depression Scale (CES-D) by Radloff (1977) is a self-evaluation tool developed by the Center for Epidemiological Studies (CES) of the National Institute of Mental Health (NIMH), based on several validated depression scales. It was developed for use in epidemiological studies of depressive symptomatology in the general population. The French version by Fuhrer and Rouillon [42] validated by Morin and colleagues (2011) was used. This questionnaire consists of 20 items, which can be grouped into four distinct subscales: depressed mood, positive affect, somatic complaints, and interpersonal relationships. The CES-D score is the sum of all these scores and ranges from 0 to 60. The higher the score, the greater the depressive symptomatology. Based on the work done by Fuhrer and Rouillon [42], the proposed threshold is 17 for men and 23 for women. Beyond these thresholds, depressive symptomatology is considered significant.

^d^STAI-Y: The State-Trait Anxiety Inventory (STAI) or Spielberg’s Form Y State-Trait Anxiety Inventory (1993) is a self-report tool for anxiety composed of two distinct scales aimed at evaluating the psychological aspects of state anxiety (STAI Y-A) and trait anxiety (STAI Y-B). The first refers to a temporary emotional state linked to a particular situation or moment and likely to appear in any individual, whereas the second refers to a more permanent or even pathological personality trait linked to the person. Each of these two scales consists of 20 items. The respondents used a 4-point Likert scale (1 to 4). The scores are therefore between 20 and 80, with higher scores indicating a higher level of anxiety. Anxiety is considered very high when it is higher than 65; high from 56 to 65; medium from 46 to 55; low from 36 to 45 and very low when it is lower or equal to 35. The French version by Bruchon-Schweitzer and Paulhan (Spielberger et al. [21],) was used in this study.

### Characteristics of the population interviewed

**Fig. 3:**
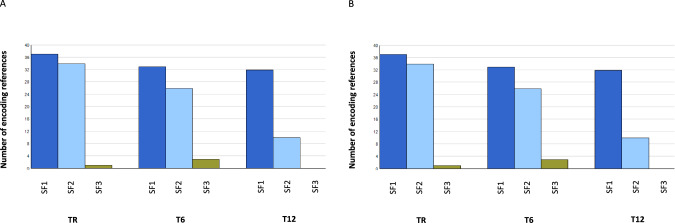
Evolution of psychological aspects *based on the analysis of* interviews according to the type of SF and the time of results. **A** Worry and anxiety; **B** Feeling at risk for participants or their family members.

Thirty parents (21 mothers and 9 fathers) of 26 patients (21 minors and 5 protected adults) with SF+ took part in a semi-structured interview (at TR, 13 with SF1, 11 with SF2 and 5 with SF3; Fig. 2). Eighty-three interviews were conducted and analyzed (29 at TR, 28 at T6, 26 at T12; 59 with mothers, 24 with fathers). Only two attributes were notable: the nature of the SF rendered and the time of analysis (Fig. 3).

### SF results

Overall, SF were identified in 47 patients.

Among SF1, 16 SF+ were reported. Variations were found in genes conferring a genetic predisposition to cancer, cardiac, hematological or metabolic diseases. For SF2, 20 SF+ were reported, of which 75% concerned *HFE gene*. For SF3, 11 homozygous or compound heterozygous variations were reported (Table 2). The family segregation is not available for three patients.

**Table 2:** Result of SF analysis and specific questionnaires at TR, T6, and T12.

Index Cases			SF1 (*n* = 16/18 returned)		SF2 (*n* = 20/22 returned)		SF3 (*n* = 11/13 returned)	
Name of gene involved			**Cardiac:** *DSG2 (1)*^*a*^*, FBN1 (1)*^*a*^*, MYBPC3 (1)*^*a*^*, MYH7 (1)*^*a*^*, RYR2*^*b*^*, TNNI3 (1)*^*a*^ **Cancer:** *BRCA2 (1)*^*a*^*, SDHB (1)*^*a*^ **Metabolic:** *LDLR (3)*^*a,*^^*c*^*, PCSK9 (1)* **Bleeding disorder:** *PROC (1)*^*a*^*, SERPINC1 (1)*^*a*^*, VWF (2)*^*a*^ **Other:** *COL3A1*^*b*^ *(1) DMD (1)*^*a*^		*CFTR (2)*^*a*^ *CYP21A2 (2)* *HFE (15)*^*a,*^^*c*^ *SMN1 (1)*^*a*^ *Pseudogene to gene CYP21A2 and SMN1*^*b*^		*CYP2C19 Hmz (6)*^*a*^^,^^*c*^ *CYP2C9 Hmz (7* + *2*^*b*^*)*^*a*^	
Identification of primary finding (PF)	With PF		7/16 (44%)		8/20 (40%)		3/11 (27%)	
	Without PF		9/16 (56%)		12/20 (60%)		8/11 (73%)	
**SPECIFIC QUESTIONNAIRE AT TR** (only in responding parents)			*N* = 19		*N* = 18		*N* = 14	
Satisfaction to have SF results (fairly to fully)			89%		94%		100%	
Worry (fairly to fully)			37%		35%		14%	
Regret requesting access to SF			0%		0%		0%	
**SPECIFIC QUESTIONNAIRE AT T6 AND T12**								
Change in lifestyle habits for the child (a little to a lot)		T6	6/16	(38%)	2/16	(13%)	1/10	(10%)
		T12	6/13	(46%)	4/17	(24%)	2/11	(18%)
Implementation of some actions of prevention for the child (food, sport…)		T6	7/16	(44%)	0/16	(0%)	1/10	(10%)
		T12	5/13	(38%)	1/17	(6%)	2/11	(18%)
Change in future projects		T6	0/16	(0%)	2/17	(12%)	0/10	(0%)
		T12	3/13	(23%)	4/17	(24%)	1/10	(10%)
Change in the vision of child’s future		T6	3/16	(19%)	4/16	(25%)	0/10	(0%)
		T12	2/13	(15%)	3/17	(18%)	2/11	(18%)
Changes in perception of the child’s body		T6	2/16	(13%)	1/16	(6%)	0/10	(0%)
		T12	2/13	(15%)	0/17	(0%)	0/11	(0%)
Change in the relationship with their child		T6	5/16	(31%)	1/16	(6%)	1/10	(10%)
		T12	3/12	(25%)	4/17	(24%)	0/11	(0%)
Change in perception of their child		T6	1/16	(6%)	2/16	(13%)	1/10	(10%)
		T12	0/12	(0%)	0/17	(0%)	0/11	(0%)
**SPECIFIC QUESTIONNAIRE AT T12**								
One year later, how do you feel about the SF results?								
Satisfaction (fairly to fully)			5/5	(100%)	4/4	(100%)	2/3	(67%)
Worry (fairly to fully)			0/5	(0%)	0/4	(0%)	2/3	(67%)

*Hmz* homozgote, *SF* secondary findings, *TR* time at results, *T6* time at 6 months after results, *T12* time at 12 months after results.

^a^Included individuals in the qualitative part.

^b^Not returned to patient (change of mind or not back for consultation).

^c^2SF.

#### First assessment: results of standardized scales and specific questionnaire before ES (QT0) (Table 1)

Most participants chose to access all three SF categories (94%; *n* = 323/340). Overall, 85% of participants had specific expectations regarding SF, mainly to implement preventive measures (77%) in order to prevent possible risks (73%), to better anticipate the future (77%), and to improve their health status (77%). Participants were ambivalent regarding the hypothetical reporting of SF for conditions with no treatment or prevention options and for SF with an uncertain status, with a tendency to wish for more results in the absence of actionability when the adults concerned are themselves questioned. However, given the small number of adults questioned, these results should be treated with great caution.

When asked how they had made their decision to access SF, 56% decided with their geneticist, 35% alone, and 17% with their partner. According the qualitative data, the choice was considered difficult for a majority of participants even though they expressed a strong motivation.

#### Second assessment (TR) and follow-up assessments (T6 and T12)

##### Medical actionability: a strong and unanimous expectation at time of report and during follow-up (Table 2)

At TR, the parents were globally satisfied by the offer to access SF results (94%), especially if a PF was found (100%). At T12, satisfaction remained high (92%). More specifically, at TR, parents whose child received a SF1 were satisfied that it would lead to better monitoring (86%) and allow them to inform their family (67%). Parents whose child received a SF2 were satisfied overall, but the reasons were diverse: to take an active role and to make responsible decisions with the available information (42%), but also to access to genetic counseling for future pregnancies (35%). The parents whose child received a SF3 were satisfied because their doctor could adapt treatments (81%), to avoid complications (56%), and because they could adopt an active role and make responsible decisions with the available information (50%).

For the interviewed parents, the medical actionability of the SF corresponded to a strong, unanimously expressed expectation that it would motivate their choice of access and confirm it after the report. Parents of children with disabilities, whose sense of responsibility is particularly strong, had a more pronounced desire to know whether their child had genetic risk factors in order to be able to better anticipate, prevent, act and protect their child.

##### Anxiety, depression, and quality of life

When considering all responding parents (whatever the PF and SF results), the analyses of the standardized scales showed that mothers tended to have significantly higher levels of anxiety and depression and lower quality of life compared to fathers, whatever the time of assessment (*p* < 10-3 for all score results). In addition, independently of the responder’s sex, having a PF was associated with increased anxiety and depression levels (+2.8 ± 1.2 points, *p* = 0.014; +3.2 ± 1.5 points, *p* = 0.036, respectively). Conversely, the fact that a SF was revealed was not associated with increased scores of anxiety and depression (pmin = 0.236). When examining specifically SF+ situations, the gap between anxiety and depression levels for mothers and fathers persisted over time (*p* = 0.037 and 0.015, respectively). Notably, the physical component summary (PCS) of the SF12 scale also appeared to be deteriorated in mothers, particularly when SF1 were assessed.

The interviews showed that SF1 were a source of worry and anguish to a greater extent and for a longer period of time than for the other SF groups (Fig. 3) *(‘As soon as you say “hypertrophic cardiomyopathy”, everything goes on alert.’ Mother, SF1, T12, MYBPC3 variant detected in her daughter)*. Moreover, the announcement of a genetic predisposition to cancer or heart disease activated representations of death *(*“*My mother’s older brother died one morning in his bed, just like that. So I asked myself the question: Could it have been hypertension that took him? We don’t know.*”*Mother, SF1, T6, SDHB variant in her and her daughter, leading to the discovery of hypertension in the mother)*.

The anxiety and anguish caused by the announcement of a SF2 was particularly apparent in some contexts (pregnancy in progress, upcoming surgery) or due to disease representations *(*“*It’s horrible. It’s a slow end. It’s witnessing the agony of a child, it’s appalling.*”*Mother, SF2, TR, CFTR, variant detected in her daughter)*, especially at the time of the results announcement, although this effect faded over time. On the contrary, the reporting of SF3 was not anxiety-provoking: “*It’s of no great consequence*” *(Mother, SF3, TR, CYP2C9)*.

Parental concerns and anxiety were maintained over time because the risk factor announced concerns the child first, but then potentially the parents themselves, and then other family members, especially siblings (“*The anguish with regard to my other children, to tell myself that I hope I didn’t give it to them too.*” *Mother, SF1, TR, DSG2 gene)*. Having to disclose the findings to the rest of the family was also anxiety-provoking, depending on the situation.

Nine out of 30 parents recalled the shock caused by the SF announcement and in a more significant and prolonged way for SF1 (“*It was announced, telling me that I was like my daughter, at high risk. I had flipped a coin. I didn’t end up on the right side of the coin. It was like a cold shower.*” *Mother, SF1, T6, BRCA2 variant carrier like her daughter)*. In retrospect, half of the parents said it would be impossible to anticipate the return of a positive SF result and its consequences.

Specific situations where the discovery of SF1 had a psychological impact have been summarized in Table 3. In three patients, psychological follow-up was implemented given the issues generated by the SF result, added to an already medically complex situation, and in a particularly precarious social situation. The SF result was not the central reason for this follow-up but it added another layer of vulnerability.

**Table 3:** Detailed presentation of the interviewees’ situations.

Primary finding presence (PF+/PF-) and type of transmission	Type of SF and transmission	Parent interviewed	Standardized scales	Family situation	Interview summary	Medical benefits	CONCLUSION
PF+, de novo	SF1 cardiac (MYBPC3), paternal inheritance	Mother	Normal	Complex familial situation, parents in the process of separating	At TR, The result of the SF was put aside, as the parents were focused on the PF. However, the SF rapidly became a source of anxiety in the days that followed, as she had to undergo surgery. Therefore, the SF was perceived as very useful, even if cardiac follow-up was poorly received by the cardiology team who were not well prepared for the issue of SF. For the mother, SF became the equivalent of saving a life. The mother anticipated that she would be a carrier because of FH of sudden death, but the variant was finally identified in the father. It was described as a shock for him, he refused cardiac screening and transmission of the information to his family members.	Patient asymptomatic at T12.	Large divergence of experience and attitude between father and mother. Psychological difficulty for the father who carries and transmits the variant.
PF-	SF1 cardiac (DSG2), maternal inheritance	Mother	Normal	Complex familial situations, foster children	At TR, the mother mentioned deaths from heart disease on her side, so “I might as well get rid of anything that could worry me and take care of it right away”. At T6, tests revealed that the mother and maternal grandmother were carriers. Their cardiac ultrasound were normal. She feared that she had transmitted the disease to her other children. At T12, tests revealed that the sister, brother, and half-sister were also carriers; the results of 2 other children are pending. She had a defensive strategy: “If [the disease] had to develop, it would have already developed. So that’s what’s really reassuring”.	None symptomatic at T12. Intensive sport practice, no intention to reduce their sports practice.	Familial context complicated. A certain denial of results
PF+, de novo	SF1 cancer (BRCA2), maternal inheritance	Mother	All high level	Divorced	At TR, the mother talked more about the PF than the SF, but mentioned her mother’s family history of cancer and deaths. The adult patient only remembered the SF. At T6, tests revealed that the mother was a carrier and that SF was inherited from her paternal side, which revealed family secrets. Two prophylactic surgeries were quickly scheduled, with anxiety on their impact on femininity. At T12, the mother verbalized major complaints and psychological distress, as prophylactic surgery had been performed but not reconstruction, which had been deprogrammed because of COVID crisis. There was a very strong impact on quality of life. She also mentioned her stress regarding the risk of her other children.	None symptomatic at T12. Prevention complicated for patient because autistic. Screening for ovarian cancer first, then breast cancer later.	Psychological follow-up suggested because of great distress, particularly at T12. Accumulation of familial vulnerabilities before the results, and serious medical and psychological consequences of the prophylactic surgery for SF.
PF-	SF1 cancer (SHDB), maternal inheritance	Mother	All high level	Single mother living alone with her child in a precarious situation, family in native country.	At TR, the comprehension of SF was difficult. She recounted a difficult life story. At T6, tests revealed that the mother was a carrier. The blood test was impossible to perform on her daughter, and she was diagnosed with hypertension. The risk for family members has been transmitted but genetic testing was impossible in her native country. At T12, it was impossible to perform a CT scan on her daughter because of ASD. SF was seen as the equivalent of saving a life, and the mother was very grateful to France and its health and social system. Her social situation remained difficult.	Hypertension in mother with introduction of physical activity and diet for weight loss	Psychological follow-up proposed because of great distress, particularly in relation to the vulnerability of the social situation and the onset of symptoms in the mother. Positive scores on all scales.
PF+, de novo	SF1 Duchenne myopathy (DMD), maternal inheritance	Mother	STAI-Y-State at TR high level	No problem	At TR, a state of shock and depression were revealed, especially linked to the announcement of the PF, but also to the SF (“the icing on the cake” “the shitty thing”). The mother evoked a strong parental responsibility and the feeling of not being given a choice (“it would be criminal” not to look for the SF). At T6, the tests revealed that the mother was a carrier. The mother described a very troubled period after the announcement. She was worried to announce results to her mother, and decided to postpone the transmission of the information. The risk concerned her daughter for their offspring, but this could be anticipated and managed. At T12, the mother was suffering from depression following the death of her father and change in career plans. The variant was finally reclassified as benign: “The chapter is closed” “Return to a normal life”.	Very high psychological impact with no medical benefit since the variant has been reclassified as benign at T12.	Psychological follow-up necessary but already in place. Psychological difficulties unrelated to the study but significant additionnal anxiety led by the SF results.
PF-	SF1 cardiac (MYH7), not maternal inheritance	Mother	EPICES, CESD, and STAI-Y-State High level	Single mother. Family in native country.	At TR, the mother was very worried about the result: “Are we going to die?” and fatalistic: “That’s the way it is, we have to accept it”. At T6, she was still waiting for the cardiology appointment, still fatalistic but confident because she “believes in her God”. The mother did not carry the variant. At T12, the son’s cardiological check-up was normal. No analysis could be done in her family in her native country. The mother was exhausted by managing her son’s severe behavioral problems.	Patient asymptomatic at T12.	A situation of social and psychological vulnerability that has been contained over time thanks to religious beliefs.
PF+, de novo	SF1 hypercholesterolemia (LDLR), maternal inheritance	Mother	STAI-Y-State at T6 and T12 high level	No problem	At TR, there was a history of familial hypercholesterolemia: the mother and maternal grandfather were already being treated with statins, and so the mother was already in a situation of anticipation and prevention, which had justified her choice to learn about SF. The result of the SF search was quite enlightening as the mother understood the genetic origin of the hypercholesterolemia. At T6, the family was strongly mobilized to improve their diet thanks to the SF diagnosis. The mother was about to undergo breast cancer surgery: she was reassured by the absence of genetic predisposition to breast cancer, “not to have passed it on!” At T12, nothing new was mentioned; the mother was taking her treatment seriously.	Positive impact of SF result on family medical care, with increased physical activity and diet	Positive medical outcome with no major psychological consequences
PF-	SF1 hypercholesterolemia (LDLR), maternal inheritance + SF3 (CYP2C19)	Father	EPICES, CESD at TR, and STAI-Y-State at TR high-level	No problem	At TR, the father was very concerned about his child’s autism. The impact of SF was minimized: “cholesterol can be treated”; he didn’t feel concerned by the pharmacogenetic SF because his son wasn’t taking any treatment. At T6 and T12, the impact of SF was still minimized, and family information was not provided, as it was not important for the father.	Patient asymptomatic at T12.	No worries from the SF. The father preserved himself by not placing much emphasis on genetics, also for PF.
PF-	SF1 bleeding disorders (SERPIN C1), not maternal inheritance	Mother	EPICES high level	Single mother	At TR, The mother was surprised with the results because her son already had several surgeries with no problem. She was not worried because it was manageable. At T6, she was waiting for the specialized hematology consultation to find out more. The mother did not carry the variant. At T12, she had understood that there was a risk of hemorrhage. She has informed her family and in particular her daughter because she suffered from heavy menstrual periods.	Patient asymptomatic at T12.	Socially vulnerable but psychologically strong. No major psychological impact.
PF+, autosomal recessive	SF1 bleeding disorders (PROC), NA (not desired by parents)	Father	Normal	2 severely disabled children	At TR, this father felt strongly responsible for his 2 severely disabled children, with the aim of “living as long as possible to be able to look after them”. He made little mention of the SF. At T6, family information was given on each family lineage At T12, he no longer talked about the SF, the difficulty of everyday life was the main concern.	Patient asymptomatic at T12.	Social vulnerability, the heaviness of everyday life left little attention for the SF
PF-	SF1 cardiac (TNNI3), maternal inheritance	Mother	CESD, STAI-Y-State and STAI-Y-trait high level	Complicated family situation, 2 severely disabled children	At TR, the mother had high expectations on the actionability of the SF, as she had non-verbal autistic twins. Her concern was to organize cardio monitoring with 2 autistic children. At T6, tests revealed that she was a carrier, which added to her anxiety as she was already being monitored for other pathologies. At T12, she expressed disappointment, anger and illusion of risk control, which in the end did not lead to reassurance but to more questioning, doubt, and concern for her daughter, notably because the cardiologist refused to test her genetically because of her young age.	Patients and mother asymptomatic at T12. Critical of the proposed cardiological follow-up.	Psychological follow-up proposed in a context of personal psychological vulnerability and the heaviness of everyday life (autistic twins).
PF+, autosomal recessive	SF1 cardiac (FBN1 Marfan), maternal inheritance	Mother and Father	Normal	No problem	At TR, the father expressed concern, a desire to speed up the examinations, and anticipated that he would be a carrier. The mother relied on medical actionability to contain the anxiety aroused by the announcement of this SF. At T6, the parents were awaiting their genetic results. At T12, the analyses revealed that the mother was a carrier, the maternal family had been informed, but all tests (genetic and echocardiography) were suspended due to COVID-19 lockdown.	Asymptomatic at T12. “We continue to live as before”.	No social and psychological vulnerability. Anxiety was control

*SF* secondary finding, *PF* primary finding, *TR* time of result, *T6* time at 6 months of result, *T12* time at 12 months of result.

##### Impact on lifestyle

In the questionnaires, the parents in SF1 revealed that there had been major changes in care and lifestyle for their child (38% at T6 to 46% at T12), including the implementation of preventive measures (e.g., sport or diet) (44% at T6 to 38% at T12).

##### Change in feelings

Although some interviewed parents said they felt serene, confident and reassured, others expressed ambivalent feelings at later points in time (for SF1 and SF2 in particular). These participants measured both the benefits of the search for SF and the associated risks *(*“*I am quite satisfied with the fact that I have an answer, and at the same time, I am a little apprehensive about what can happen afterwards, about anesthesia, possible surgery…*” *Father, SF1, PROC gene, T12)*. The importance of medical and psychological support before and after the decision-making process also emerged in some parents’ later comments.

After the reporting of results, the carriers acquired a new status of: “being at risk” [24].They no longer felt fully healthy but they were not yet ill. This liminal stage was drawn out by uncertainty and doubts. We observed a growing and sustained state of confusion over time for individuals who had SF1 results more than for SF2 and SF3 (Fig. 3) (“*There was something we didn’t know, and now we have this risk in the family. We live with it, it’s something we don’t see but we live with that risk in the family. Now we know it’s there. Before, we didn’t know that we had something like that in our body” mother, SF1, SDHB gene, T6)*.

Later on, for SF2 and SF3, some participants could not remember the results (7/29), indicating that they were not particularly concerned about SF that had a limited effect. For others (10/30), there was confusion with the PF, raising questions about the difficulty of understanding such complex results.

Finally, all of the parents who responded to the questionnaires and 28/30 interviewed parents said that they had no regrets about accessing SF results (*’It still allows you to be able to protect and preserve. Make sure everybody’s okay. So no, I have absolutely no regrets. And tomorrow if I’m asked to do it again, I’ll do it 100%’ Mother, SF1, DSG2 gene, T12)*. Twelve parents out of 30 put forward the fact of being able to anticipate thanks to the SF *(“For this Marfan disease I’m not more worried than that because there’s a very correct follow-up we’ll say, we’re well supervised, everything has been explained to us correctly.” Mother, SF1, FBN1 gene, T6; “It’s useful, afterwards we’re lucky, we’ve come across a disease that’s easy to treat, and what’s more easy to detect, so it’s interesting to have had the information all of a sudden.” Mother, SF2, HFE gene, T12*”).

## Discussion

Our study design was developed in an attempt to asses, using a forward-looking approach, the many issues raised by SF in a since only the return of IF to patients is currently authorized in routine care by bioethics laws in France, while SF is still prohibited outside the research context [25]. Access to SF is seen as a change of clinical paradigm because it offers patients an opportunity to access results beyond their initial request. Furthermore, this offer is contrary to the principles and protocols that have been implemented worldwide to best manage sensitive requests for presymptomatic diagnoses. Nevertheless, because this is a major issue of interest in the development of genomic medicine, research studies have particularly been encouraged to discuss the evolution of positions in the future. In some respects, the results gathered in this study of SF can also be transposed to IF.

For the FIND study, we chose a mixed design combining standardized anxiety, depression, and quality of life scales with specific questionnaires studying the psychosocial impact (satisfaction, worry, and changes in life habits) and qualitative interviews allowing us to refine these different aspects. We felt this approach was essential if we were to properly interpret the results in families that have been severely affected by a rare disease. The collection of this data again at 6 and 12 months allowed us to assess the mid-term effect of the reporting of results. We showed that, while the reporting of SF results had little influence on the standardized scales, but it could have real repercussions at the individual level, particularly when a predisposition to late-onset actionable diseases was revealed. Although we expected the SF1 to have a greater psychological impact than the SF2 and SF3, we found that occasionally there were psychological effects related to particular situations, particularly among women. This study confirms that quantitative data alone are not a good marker of the impact of the SF results seeing as they obtained some answers but without any reference to the subject’s personal history. Therefore, a qualitative assessment of the interviews was essential for identifying how individuals were affected by SF in a contextualized and understandable way. The qualitative study suggested, for example, that surprise and shock may be linked to an impossible anticipation, since they were not sufficiently psychologically prepared to get these results.

The discourse of a mother who has breast cancer but no genetic predisposition to breast cancer identified in her child *(“not to have passed it on!” Mother, SF1, LDLR gene, T6)* alerts us to the sometimes unrealistic expectations (knowing or eliminating all risks), with the risk of false reassurance of negative result. Indeed, a false-negative result could lead patients at risk of optimism bias [26], to false reassurance [27].

Some of our results are consistent with those of previously published real-life studies (3/6 from North America) evaluating the impact on patients in the short to mid-term (Table 4) [28–33]. Only one study had a mixed design; the others were quantitative or qualitative. In general, wanting to access SF was motivated by medical actionability [28]. Our study showed that the lifestyle repercussions and parents’ perception of their children was different in SF1 (actionable late-onset disease) and in the two other groups. Consequently, despite the psychological and social issues, most studies, including ours, found that no or few patients regretted accessing their SF results [17, 29–34].

**Table 4:** Review of other real-life studies.

REFERENCES (Country)	METHODOLOGY			RESULT				CONCLUSIONS
	Study design	Study population and number of participants	Modality to present SF/IF	Choice of access to SF (%, reason and risk)	Type of SF/IF and % of SF/IF identified	Experience of the announcement	Psychological impact	
QUANTITATIVE STUDY								
Wynn et al. [31] (USA)	Medium-term study; Questionnaires at inclusion and between 1 and 12 months after announcement of results for the ES group.	*n* = 192: 107 patients with a history of breast cancer or congenital heart defect or developmental disorder (ES group—real life group) and 85 patients awaiting exome (No ES group—hypothetic group).	Recruitment via invitation letter and follow-up phone call	Desire to have the SF: 76% initially, then 65% after the information consultation. Choice based on subjective experiences rather than medical categories.	SF in large list of predisposing genes, including low penetrance genes (Alzheimer, CMT neuropathy, cancer, cardiomyopathy, arrhythmia, hemochromatosis, metabolism, coagulation) 37% (40/107)	High level of satisfaction; feelings of empowerment from having the knowledge.	Anxiety and depression levels similar to the general population in the ES group with or without SF (increased anxiety in the group without ES, not significant) more use of coping methods (based on emotion management and problem management in the ES group with SF)	*No differences in psychological and social measures between the beginning and end of the study, or between the ES group (real-life group) and non-ES group (hypothetic group).* *No regrets about participation*
QUALITATIVE STUDIES								
Hart et al. [29] (USA)	Medium-term study; Interviews at 4 months after announcement of SF results	*n* = 6240 exomes in patients with cancer or rare diseases *n* = 18 interviewed with SF: 10 adult patients, 8 parents of minor patients	NA	NA	SF in ACMG list of 56 genes (V1) 1.7% (74/6240)	Surprise: 15/18; Surprised but not shocked because possibility of finding SF mentioned beforehand Inter-individual variability: some ignored these results; others have described it as a “shock, were scared and have to work hard to avoid thinking about it”.	Minimal psychosocial effect according to authors Modification of the insurance contract: 3/18	Moderation of initial reactions over time Feeling of relief and gratitude for accessing the SF Minimal psychosocial impact *No regrets*
Ormondroyd et al. [32] (UK)	Medium-term study; Interviews between 6 and 12 months after the announcement of the SF results	*n* = 7203 genomes in patients with rare diseases. *n* = 4 interviewed with SF	Invitation by letter, followed by meeting with multidisciplinary team who present study rationale, possible outcomes, consequences of SF and presence of an ICC gene variant for the participant and relatives	Agreeing to participate in the study out of altruism and for family and personal reasons	SF in hereditary heart diseases genes 0.61% (44/7203)	Anxiety for one symptomatic patient with family history. Experience modulated by the history of the primary disease.	Concern about the risk to their already disabled children. Fear of not being able to continue working for a patient. Frustration and anxiety due to the long waiting time for cardiac explorations and because of the refusal of genetic tests by certain family members	Satisfaction of the participants *No regrets*
Schoot et al. [28] Schoot et al. [43] (The Netherlands)	Medium-term study; Interviews several months after the announcement of results.	16,482 index patients received clinical ES). *n* = 20 interviewed; 10 parents with oncological IF and 10 with cardiac IF.	Short information in prescription consultation	PF/IF confusion (parents think no test if no IF search)	Medically actionable IFs and carrier status of a recessive disease; all discussed in a multidisciplinary team IF in 0.58% (95/16,482)	Some participants forgot about the possibility of discovering IF Psychological impact emphasized in all cases: “overwhelmed, upset, shocked. (Initial feeling that faded over time when the actionability of IF was better perceived and daily life resumed).	Decreased ability to memorize information due to the emotional dimension Questioning about being sick or healthy.	Relevance of receiving information through in-depth *pre-and post-test counseling and medical follow-up consultations.* *Influencing factor of psychological, physical, and financial consequences: actionability of the IF, understanding, pre-test health status and social context*
Cheung et al. [33] (Canada)	Long-term study; Interview at 1.3–3.9 years	500 families of children with undiagnosed suspected genetic conditions *n* = 12 interviewed parents with IF	Information by a genetic counselor as per the recommendations by the CCMG	IF acceptance by 90% Curiosity, desire to know everything in order to act accordingly Influence of personal beliefs and values + having a child with a disease;	IFs related to highly penetrant conditions, reviewed on a case-by-case basis by a multidisciplinary study team 2.3% (21/901)	Surprise at the announcement, anxiety, worry, and nervousness for some patients, especially if pharmacological, oncological, or cardiac IF	According to personal and family history and health beliefs	*Different impacts depending on the type of IF and family history* *No regrets*
MIXED STUDIES								
Sapp et al. [30] (USA)	Medium-term study; Interviews and questionnaire at 4 months after announcement of results	*n* = 1197 exomes in patients with cancer or rare diseases. Interview: *n* = 13: 10 adults, 3 parents of minor patients with SF Questionnaire: *n* = 107 patients without SF	NA	Confusion between primary diagnosis and SF = 17%	SF in ACMG list of 56 genes (V1) 1.9% (14/1,197)	With SF: Surprise in 10/13, initial shock and disbelief then relief and gratitude with hindsight Without SF: Reassured but also disappointed when there was familial history of the sought diseases	Concern for themselves or their children = 5/13	*Minimal psychosocial impact.* *11/13 no regrets*

*ES* exome sequencing, *SF* secondary finding, *IF* incidental finding, *PF* primary finding, *TR* time of result, *T6* time at 6 months of result, *T12* time at 12 months of result, *NA* not available, *ICC* inherited cardiac conditions, *CCMG* Canadian College of Medical Geneticists.

Despite positive feedback overall, the nature of the announced SF appeared to be a determining factor in the intensity of parental worries and anguish, especially depending on the nature of the representation caused by the result (representation of death associated with cancer and cardiovascular risks). Similar to our study, previous studies showed that there is not a severe psychosocial impact for participants [29–31], and that the individual’s previous state of anxiety, history and the type of SF seem to be the factors that most influence how they experience the announcement [28, 29, 33, 35, 36]. Interestingly, the crosscheck of quantitative and qualitative data confirmed that the initial psychological status was an important prognostic indicator for the participants’ reaction when the genetic results for SF were reported. Accordingly, parents with history of depression and/or a high-level of anxiety had a higher risk of psychopathological consequences following the announcement of a SF for their child [31].

Also, we believe that it will be essential to provide additional support for individuals in situations identified as at-risk. With the current resources, if SF were to be offered to all persons undergoing ES/GS, it would be impossible to provide specific clinical consultations and systematic psychological support in each case. However, at-risk patients should be identified and psychological support offered if the patient is in distress.

Otherwise, this study revealed some SF actionable in adulthood among minors, providing relevant information for the children’s parents/families. At the same time, due to the organization of French health care, and without a national shared medical record, we must anticipate that contacting the proband in adulthood could be challenging. In addition, when Feinberg [37] insisted on the importance of preserving the “right for a free future”, he underlined that it is necessary to ensure the quality of the early child-parent relationship while protecting the child from too much knowledge. Richer and Laberge proposed that the list of accessible SF could be revised to contain only diseases actionable from childhood [38]. We recognize that this was a limitation in the design of the FIND study, therefore our team is now conducting a new study, ancillary to the DEFIDIAG study (DEFIDIAG-DS), proposing trio GS in patients with undiagnosed ID [39]. In DEFIDIAG-DS, the search for SF (ACMG list version 2) is offered to parents but not to their child. Results will be expected in the future.

Finally, the search for SF can be similar to a pre-symptomatic testing process without information regarding family history. Studies on this subject can help us to establish a framework by better measuring the psychosocial consequences of reporting SF [35, 36, 40]. To extend our understanding of this subject, we have prolonged the follow-up for patients in whom SF was identified in the FIND study over a period of 10 years, which should allow us to better evaluate the long-term effects and to measure the consequences in terms of access to care and on the family as a whole.

In conclusion, using data obtained from standardized scales, questionnaires, and interviews, the FIND study revealed that the diagnostic reporting of a genetic SF result is not a trivial matter and that there are mid-term consequences on psychological health, care pathways, and lifestyle. Although participant satisfaction was high and, in most cases, serious incidents were not observed, care should be taken to avoid putting patients in situations of vulnerability and, as much as possible, patients need to be guided and supported when choosing to generate and access this type of presymptomatic screening, especially after the results are delivered. This study, thanks to its mixed methodology and the fact that it took into account the psychological state and anxiety of patients prior to the reporting of results, can be used as a point of reference for countries that have not yet decided on the question of offering access to SF in patients undergoing ES/GS.

### Supplementary information

Supplementary material

## Data Availability

Contents of specific questionnaires and interview grids available on request URL  for websites referenced in the article : https://www.agence-biomedecine.fr/IMG/pdf/20200107_rbp_donnees_additionnelles_dv.pdf. https://www.ffgh.net/index.php/presentation/documents-ffgh. https://www.genome.gov/health/Genomics-and-Medicine
