# Supplementary material for: Expectations, needs and mid-term outcomes in people accessing to secondary findings from ES: 1st French mixed study (FIND Study)
Source: Eur J Hum Genet. 2024 May 27;32(9):1166–83. doi: 10.1038/s41431-024-01616-9 (PMC11368951; doi:10.1038/s41431-024-01616-9)
Supplement: Supplementary file 1 — Supplementary material [file 41431_2024_1616_MOESM1_ESM.docx]

***Supplementary Table 1: List of genes***

| Group of genes | | Genes |
| --- | --- | --- |
| SF1  122 actionable genes responsible for a predisposition to a later-onset disease  (expected in 4% of the study population according to the literature). | 59 genes of the minimum ACMG list (Biesecker et al. 2017) | **Adult-onset only**  Autosomal dominant inheritance: *BRCA1, BRCA2, MLH1, MSH2, MSH6, PMS2*  Autosomal recessive inheritance: *MUTYH*  **Pediatric and adult-onset**  Autosomal dominant inheritance: *ACTA2a, ACTC1, APC, BMPR1A, CACNA1S, COL3A1, DSC2, DSG2, DSP, FBN1, KCNH2, KCNQ1, LMNA, MEN1, MYBPC3, MYH11, MYH7, MYL2, MYL3, NF2, PCSK9, PKP2, PRKAG2, PTEN, RB1, RET, RYR1, RYR2, SCN5A, SDHAF2, SDHB, SDHC, SDHD, SMAD3, SMAD4, STK11, TGFBR1, TGFBR2, TMEM43, TNNI3, TNNT2, TP53, TPM1, TSC1, TSC2, VHL, WT1*  Semidominant inheritance: *LDLR, APOB*  Autosomal recessive inheritance: *ATP7B*  X-linked recessive inheritance: *GLA, OTC** |
|  | 59 additional genes predisposing to preventable and treatable conditions that may develop later in adulthood (Dorschner et al., 2013) | **Adults-onset only**  Autosomal dominant inheritance: *CDH1, EPCAM, FH, FLCN, PROC, PROS1, SERPINC1, SGCD*  Autosomal recessive inheritance: *COQ2, HAMP, HFE-rs1800562*, HFE2*  **Pediatric and adult onset**  Autosomal dominant inheritance: *ACVRL1, CACNA1C, CACNB2, CDC73, CNBP, DMPK, ENG, GCH1, GPD1L, HCN4, HMBS, KCNE1, KCNE2, KCNE3, KCNJ2, KIT, MET, MLH3, MYLK, PDGFRA, PLN, PRKAR1A, PTCH1, RBM20, SCN1B, SCN3B, SMARCB1, TGFB3*  *Autosomal recessive inheritance: BCHE, BLM, CASQ2, COQ9, CPT2, F5b-rs6025/F5b, GAA, IDUA, LDLRAP1, PAH, PCBD1, PTS, QDPR, SERPINA1, SLC25A13, SLC37A4, SLC7A9*  X-linked recessive inheritance: *DMD, EMD* |
|  | 4 additional genes predisposing to coagulation disorders | *F2 (rs1799963), F8, F9, VWF* |
| SF2  114 genes with implications for genetic counseling  (expected in 10% of the study population according to the literature). | 4 genes with a high frequency of heterozygotes in France, accessible to family screening | *CFTR* variants: rs199826652, rs113993960, rs113993959, rs80034486, rs76713772, rs80224560, rs74597325, rs75527207, rs79660178, rs75096551, rs77188391, rs121908745, rs76151804, rs77932196, rs74767530, rs77010898, rs121908761, rs121908799, rs121908747, rs397508266, rs75961395, rs74503330, rs121909011, rs78655421, rs397508393, rs267606722, rs78756941, rs121908744, rs74551128, rs77284892, rs121908769, rs75039782,  *HFE* variant: rs1800562*, *SMN1* deletion, *CYP21A2* |
|  | 110 genes involved in X-linked intellectual disability | *ACSL4, AFF2, ALG13, AP1S2, ARHGEF6, ARHGEF9, ARX, ATP6AP2, ATP7A, ATRX, ATXN3L, BCOR, BRWD3, CACNA1F, CASK, CCDC22, CDK6, CDKL5, CLCN4, CLIC2, CNKSR2, CUL4B, DCX, DDX3X, DKC1, DLG3, DP71, EIF2S3, FANCB, FGD1, FLNA, FMR1, FRMPD4, FTSJ1, GDI1, GK, GPC3, GRIA3, HCCS, HCFC1, HDAC8, HPRT1, HSD17B10, HUWE1, IDS, IGBP1, IKBKG, IL1RAPL1, IQSEC2, KDM5C, KIAA2022, KLF8, KLHL15, L1CAM, LAMP2, LAS1L, MAOA, MBTPS2, MECP2, MED12, MID1, MSL3, NAA10, NDUFA1, NHS, NLGN3, NLGN4X, NSDHL, OCRL, OFD1, OPHN1, OTC*, PAK3, PCDH19, PDHA1, PHF6, PHF8, PLP1, PORCN, PQBP1, PRPS1, PTCHD1, RAB39B, RBM10, RLIM, RPS6KA3, SHROOM4, SLC16A2, SLC35A2, SLC6A8, SLC9A6, SMC1A, SMS, SOX3, SYN1, SYP, TAF1, TIMM8A, TMLHE, TSPAN7, UBE2A, UPF3B, USP27X, USP9X, WDR45, ZDHHC15, ZDHHC9, ZMYM3, ZNF711, ZNF81* |
| SF3  2 actionable genes with compound heterozygous or homozygous pharmacogenomic variants  (expected in 12% of the study population according to the literature). | | Short list of pharmacogenetic variants of clinical interest studied in the framework of the study, which may be of interest with regard to a possible antiepileptic or psychotropic treatment: *CYP2C19* and *CYP2C9* |

* Commonly present in actionable genes leading to predisposition to a later-onset disease and in the genes for genetic counselling

*Legend: Each group has a different impact that was of interest to study and are part of the SF sets that could be proposed.*

*Only ACMG class 4 and 5 variants could be returned.*

*The inclusion of patients heterozygous for the p.Cys282Tyr variant of the HFE gene in group 2 was debated seeing as family cascade studies are no longer necessarily recommended in families where a p.Cys282Tyr homozygote is detected.*

***Supplementary Table 2: Characteristics of index cases (n=340)***

| Sex (338) | Male | | 191 (56%) |
| --- | --- | --- | --- |
|  | Female | | 147 (44%) |
| Age (340) | Fetus | | 10 (3%) |
|  | Less 18 years old | | 248 (73%) |
|  | Over 18 years old | | 79 (23%) |
|  | Deceased | | 3 (1%) |
| Type of disease (335) | Intellectual disabilities (ID) | ID associated with polymalfomative syndrome | 181 (74%) |
|  |  | ID associated with other NDD | 3 (2%) |
|  |  | Isolated ID | 55 (22%) |
|  |  | *Total* | *242 (71%)* |
|  | NDD without ID | | 20 (6%) |
|  | Polymalformative syndrome without ID | | 64 (19%) |
|  | Other (neuromuscular, deafness or metabolic diseases) | | 9 (4%) |
| Other person with the same condition in the family (337) |  | | 50 (14.8%) |
| Genetic investigations (335) | Previous exploration(s) | | 249 (74%) (221 with chromosomal microarray) |
|  | diagnostic wandering time (Median (minimum maximum) | | 3 years (min 0 month , max 27 years) |

NDD: Neurodevelopmental disorders

***Supplementary Table 3: Results of questionnaire and interview of parents without SF***

|  | Number of participants | Conclusions |
| --- | --- | --- |
| Questionnaires | 380 | The analysis of the questionnaires of parents who had no SF showed that half of the parents were reassured (46% if no PD was found and 55% if a PD was found). It should be noted that more than 80% of parents wanted to receive a SF if the relevant scientific knowledge changed. |
| Interview at time of results | 8 | The analysis of interviews showed that some parents had confusion between PD and SF, and sometimes accepted the search for SF because they thought it would increase their chances of identifying a PD (Mother PD+: *Yes, but I mean that was the risk we had to take to get the results anyway. After that, there was no question, we absolutely had to know what he had, so we had to do it.*).  For those who remembered what a SF was and what type of SF could be identified, the search for SF was described as an opportunity to prevent and anticipate late-onset disease. The majority was relieved not to have had a SF identified (Father PD+ : *So it's quite clear that if something really serious had happened, I'm not telling you, perhaps it would have caught our attention at the time, but not out of fear, in any case ;* Mother PD+ *: We're glad we made the request, because now there's no secondary data, so we're reassured.*)*.* |

*SF: Secondary Finding ; PD : Primary Diagnosis*
